# Adiponectin levels decrease independently of body mass index and diabetes type after the normalization of hyperglycemia

**DOI:** 10.3906/sag-1903-35

**Published:** 2020-04-09

**Authors:** Nalan METİN AKSU, Duygu YAZGAN AKSOY, Meltem AKKAŞ, Nese ÇINAR, Fatma UÇAR, Bülent Okan YILDIZ, Aydan USMAN

**Affiliations:** 1 Department of Emergency, Faculty of Medicine, Hacettepe University, Ankara Turkey; 2 Department of Internal Medicine, Faculty of Medicine, Acıbadem Mehmet Aydınlar University, İstanbul Turkey; 3 Department of Internal Medicine, Faculty of Medicine, Muğla Sıtkı Koçman University, Muğla Turkey; 4 Department of Biochemistry, Yıldırım Beyazıt Dışkapı Research and Training Hospital, Ankara Turkey5; 5 Department of Internal Medicine, Faculty of Medicine, Hacettepe University, Ankara Turkey

**Keywords:** Hyperglycemia, adiponectin, body mass index

## Abstract

**Background/aim:**

Acute hyperglycemia is generally a frequently encountered condition in the emergency department (ED), because it is seen as a complication of diabetes mellitus (DM). In this study, we aimed to detect the change in adiponectin levels during acute hyperglycemic states and after normalization of blood glucose with insulin treatment.

**Materials and methods:**

Forty-eight patients over the age of 18 years who were admitted to the ED with acute hyperglycemia were included in the study. Serum samples were taken from patients on admission and 6 h after the normalization of blood glucose with insulin treatment, and adiponectin levels were measured in both samples.

**Results:**

There were 21 female and 27 male patients with a median age of 58.7 ± 18 years. All patients’ blood glucose levels were normalized with insulin treatment according to international recommendations. Serum adiponectin levels decreased significantly after the normalization of blood glucose in the whole group. Adiponectin levels decreased from 28.9 ± 16.5 to 12.1 ± 10.9 μg/mL (P < 0.0001) in the whole group. This decrease was independent of diabetes type and body mass index.

**Conclusion:**

Normalization of blood glucose in patients with hyperglycemia caused a decrease in adiponectin levels, independent of diabetes type and/or body weight in an acute emergency setting. Inhibited upregulation of adiponectin secretion and/or blunted suppressive effect of insulin due to hyperglycemia or exogenous insulin administration may have caused the decrease in adiponectin levels.

## 1. Introduction

Diabetes mellitus (DM) prevalence is increasing worldwide [1]. Hyperglycemia is a frequently encountered condition of patients with DM who are admitted to the Emergency Department (ED) [2].

Adiponectin is an adipokine which was initially thought to be secreted only from adipocytes, but it was later proven that adiponectin is also secreted from osteoblasts, liver parenchymal cells, myocytes, epithelial cells, and placental tissue [3,4]. Adiponectin is a major regulator of glucose metabolism with insulin-sensitizing properties; thus, low levels of adiponectin are associated with diabetes [5]. 

Adiponectin circulates in the concentration range of 3–30 μg/mL in healthy individuals. The clearance of adiponectin is primarily mediated by the liver. It has a surprisingly rapid turnover. The half-life of adiponectin is reported to be between 75 and 150 min. The serum half-life is reduced in patients with type 2 DM and it may be even shorter in patients with large fat cells and poor diabetes control [6,7]. Adiponectin levels are positively associated with insulin sensitivity. The glucose-lowering effect of adiponectin is primarily mediated by suppressing gluconeogenesis or glycogenolysis, and it may also be mediated by upregulation of Peroxisome proliferator activated receptor α (PPARα) [4]. 

Adiponectin is also known as an antiinflammatory hormone [8]. It maintains metabolic homeostasis, and higher levels of adiponectin are associated with lower type 2 DM risk [9,10]. Adiponectin, with its insulin-sensitizing, antiatherogenic, antiapoptotic, and antiinflammatory effects, may play a role in future therapies for obesity, type 2 diabetes, and atherosclerosis [4]. 

The effect of acute hyperglycemia on adiponectin levels is less studied [3,11]. We evaluated the effect of the normalization of blood glucose levels on adiponectin in patients admitted to the ED with acute hyperglycemia.

## 2. Materials and methods

### 2.1. Patients 

This study was approved by the ethical board of Hacettepe University (HEK 09/177-107). Forty-eight adult patients admitted to the ED with hyperglycemia (blood glucose of ≥300 mg/dL) were included in the study. Written informed consent forms were obtained from all patients. Demographic features (age, sex), height, weight, and type of diabetes were recorded. 

### 2.2. Methods

All patients were treated according to international recommendations for hyperglycemia, with appropriate amounts of intravenous fluid and intravenous crystalline insulin infusion. Euglycemia was reached within 6–12 h. Serum samples were taken from the patients on admission and at 6 h (which was chosen as an optimum time for half-life of adiponectin) after normalization of blood glucose to measure adiponectin levels. Adiponectin levels were measured by Biovendor Human Adiponectin ELISA. Results are given as μg/mL.

### 2.3. Statistical analysis

Statistical analyses were carried out using SPSS 15.0 for Windows (SPSS Inc., Chicago, IL, USA). Numerical variables are shown as mean (range), and categorical variables are shown as frequencies and percentages. The Mann–Whitney U test and Kruskal–Wallis test were used to determine differences in numerical variables between the groups, and the chi-square test was used to determine differences between categorical variables. P ≤ 0.05 was considered to be statistically significant.

## 3. Results

The study included 48 patients (21 female and 27 male), with a median age of 58.7 ± 18 years. Clinical and laboratory parameters are presented in Table 1. Thirty-one patients had type 2 DM, 10 patients had type 1 DM, and 7 patients were diagnosed with diabetes at that particular admission. 

**Table 1:** The distribution of the patients according to BMI, sex, and previous DM.

BMI (kg/m^2^)	Normal (<24.9)	Overweight (25–29.9)	Obese (>30)
N	16	20	12
Sex	3 F/ 13 M	9 F/11 M	9 F/3 M
Previous DM	11 (68.8%)	19 (95%)	11 (91.75%)

BMI: Body mass index; DM: diabetes mellitus. Data are presented as n (%) or mean ± SD.

Serum adiponectin levels decreased significantly after the normalization of blood glucose in the whole group. Adiponectin levels decreased from 28.9 ± 16.5 to 12.1 ± 10.9 μg/mL (P < 0.0001) in the whole group. The decrease in adiponectin persisted when evaluated according to Body Mass Index (BMI) and type of DM (Tables 2 and 3). 

**Table 2:** The change in adiponectin levels before and after treatment according to BMI.

BMI (kg/m^2^)	Number (n)	Adiponectin (μg/mL)		
		Before treatment	After treatment	P
Normal (<24.9)	16	26.4 ± 17.5	13.3 ± 13.0	0.006
Overweight (25–29.9)	20	34.6 ± 14.2	13.3 ± 11.9	<0.001
Obese (>30)	12	24.4 ± 17.1	8.9 ± 5.5	0.005

**Table 3:** The change in adiponectin levels before and after treatment according to diabetes type.

Diabetes type	Adiponectin (μg/mL)		
	Before treatment	After treatment	P
Type 1	28.8 ± 17.3	10.5 ± 8.2	0.007
Type 2	26.3 ± 18.4	8.7 ± 7	<0.001

## 4. Discussion

The results of the present study demonstrated that adiponectin levels decreased after the normalization of blood glucose in patients with hyperglycemia. This was not dependent on diabetes type and/or body weight. Studies that evaluate the effects of acute hyperglycemia are rare. This is the first study that has demonstrated a decrease in adiponectin levels after the correction of hyperglycemia.

Aso et al. investigated the effect of acute hyperglycemia after oral glucose load in healthy subjects on total and high-molecular-weight (HMW) adiponectin. HMW adiponectin decreased significantly at 120 min after oral glucose load [12]. In another study, after an acute glucose load test, HMW adiponectin decreased in patients with normal glucose tolerance tests and those with impaired fasting glucose (fasting blood glucose levels over 100 mg/dL), but there was no change in patients with impaired glucose tolerance (glucose levels of 140–200 mg/dL 2 h after glucose challenge) and diabetes (glucose levels over 200 mg/dL 2 h after glucose challenge). Percentage change in adiponectin was negatively correlated with serum insulin but not glucose levels. This change is explained by an increase in insulin levels, which were less prominent in patients with impaired glucose tolerance and diabetes [13]. 

Koniari et al. reported that glucose loading increased adiponectin levels in healthy and impaired glucose tolerant patients. This response was significantly lower in diabetic patients. They stated that acute hyperglycemia is a stress factor that upregulates adiponectin secretion. The absence of this upregulation in the diabetic group is explained by the presence of lower adiponectin levels in diabetics [14].

In a study by Siervo et al., after oral glucose tolerance tests, adiponectin levels decreased both in healthy patients and in those with metabolic syndrome [15]. Dullaart et al. evaluated the effects of insulin secretion on adiponectin levels with usage of a hyperinsulinemic clamp in type 2 diabetic patients and healthy subjects. Insulin lowered the adiponectin levels in patients with type 2 DM; however, it did not change the levels in healthy subjects [16]. Insulin receptor dysfunction is associated with increased circulating adiponectin. Insulin directly suppresses adiponectin secretion from the marrow’s adipose tissue [17]. In another study, in which the separate and combined effects of hyperglycemia and hyperinsulinemia on different markers were evaluated, adiponectin increased by euinsulinemia-hyperglycemia clamp. Adiponectin decreased in the states of hyperinsulinemia-hyperglycemia, and with the hyperinsulinemia-euglycemia clamp. Hyperinsulinemia is thought to prevent the effect of hyperglycemia in increasing adiponectin [11]. 

In this study, we demonstrated a decrease in adiponectin levels after correction of hyperglycemia with insulin treatment. This is the first clinical study that has demonstrated the effect of correction of acute hyperglycemia on adiponectin levels. Adiponectin is known to inversely correlate with body weight and abdominal obesity [18]. The decreases in adiponectin levels were independent of BMI in our study. Although visceral obesity, which is a main contributor of type 2 diabetes, has a major role in the secretion of adipokines, adiponectin levels were also reported to change and affect outcomes in those with type 1 DM [19,20]. The decrease in adiponectin was not affected by diabetes type in our study.

This change may be due to an inhibited upregulation of adiponectin secretion and/or a blunted suppressive effect of insulin due to hyperglycemia or exogenous insulin administration may have caused the decrease in adiponectin levels.

The insulin-sensitizing action of adiponectin is primarily due to decreased hepatic gluconeogenesis and it increases glucose transport in the muscles. Adiponectin mediates antidiabetic effects via direct metabolic actions, by improving insulin sensitivity, and, as recently demonstrated, by playing an important role in the stimulation of autophagy [21,22]. 

Our sample size was limited in terms of patients with different background disease profiles. This might have influenced the adiponectin levels. Scientists still need to do further studies to address the puzzling cross-talk between adiponectin and other metabolic factors.

In conclusion, adiponectin decreased after the correction of hyperglycemia in an emergency setting. This decrease was independent of diabetes type and/or body weight. Acute hyperglycemia, which upregulates adiponectin secretion and/or exogenous insulin administration, may have caused the decrease in adiponectin levels. Adiponectin is a promising adipokine in the treatment of many diseases; however, perplexing factors in both secretion and metabolism mandate, before it can be confidently used as a marker and/or a therapeutic target.

## Declaration of conflict of interest

The authors report no conflicts of interest. 

## Source of support 

This work was supported by the Hacettepe University Scientific Research and Developmental Office (010D02101002).
